# The neuroprotective role of citicoline treatment in glaucoma – 6 months results of a prospective therapeutic trial

**Published:** 2019

**Authors:** Iulia Chițu, Liliana-Mary Voinea, Sânziana Istrate, Alexandra Vrapciu, Radu Constantin Ciuluvică, Ruxandra Tudosescu

**Affiliations:** * Emergency University Hospital of Bucharest, Romania; ** ”Carol Davila” University of Medicine and Pharmacy Bucharest, Romania; *** ”Regina Maria” Private Clinic, Bucharest, Romania

**Keywords:** glaucoma, citicoline, neuroprotection

## Abstract

**Objectives.** Neuroprotective treatment, including citicoline, is a new perspective in glaucoma management, having the role of progression delay. The purpose of the present study was to observe the evolution of the different parameters in patients with glaucoma treated with citicoline.

**Methods.** 22 patients with GPUD were enrolled in the study, and they received oral citicoline in addition to the ocular hypotensive therapy. Investigations were performed at the beginning of the current study, then at 3 months and 6 months, and included, besides full ophthalmologic checkup and IOP determination, optic nerve and RGCs OCT, and visual evoked potentials, pattern and flash. The data we obtained were statistically analyzed with the SPSS (Microsoft) program.

**Results.** The outcomes of the study following VEP wave analysis indicated variations in P100 wave amplitude, but after 6 months period, an increase was found. Also, the P2 wave amplitude recorded statistically insignificant variations. The increase in P2 latency at 6 months was noted as statistically significant. Negative correlations were also met between the thickness of the RGC layer and the P100 latency, but also between the amplitude and the latency of this wave. At 6 months, a positive correlation between the RGC layer and the P100 amplitude was observed. The RNFL thickness at the optical disc had higher values at the 6 months visit, it was statistically significant, and a slight increase in the thickness of the RGC layer between successive visits was noted. These might be an examination artifact because clinically they are not possible. The RNFL thickness showed a positive correlation with the amplitude of P100 and P2 waves.

**Conclusions.** The study of the parameters and their correlations demonstrated that citicoline had positive effects in glaucoma on certain aspects, data confirmed by literature.

## Introduction

Glaucoma is as a group of progressive multifactorial optic neuropathies in which the optic nerve excavation increases as a result of retinal ganglion cells (RGC) loss, resulting in the permanent change of the visual field. It is the second cause of irremediable visual loss [**1**].

The leading risk factor for glaucoma is high intraocular pressure, but sometimes the decrease of intraocular pressure through hypotensive therapy may be insufficient and glaucoma may progress. Therefore, new adjuvant treatments, such as neuroprotectors, have been proposed to prevent, delay, or reduce the loss of neuronal cells, precisely of the RGC, by acting on cells undergoing apoptosis [**2**]. Along with the mechanical effect of intraocular pressure, injury to the optic nerve can also be determined by compromising its vascularity or by combining the two mechanisms [**3**].

Citicoline (cytidine-5'-diphosphocholine) is a pure endogenous compound acting as an intermediary product in the synthesis of membrane phospholipids, such as phosphatidylcholine, and can also increase the level of certain neurotransmitters in the central nervous system [**4**]. After exogenous administration, it is hydrolyzed and dephosphorylated, forming cytidine and choline, both of which will resynthesize the citicoline inside the neuronal cells. In a previous article, the authors have also described the positive effect of citicoline in Alzheimer’s disease, stroke, Parkinson’s disease and amblyopia [**5**].

Clinical and experimental studies have demonstrated the protective effect of citicoline on RGC by the antiapoptotic effect, thus opposing the thinning of the retinal nerve fiber layer, and thus causing a better transmission of the nervous impulse through the visual pathways [**6**]. Improvement of nerve impulse transmission was determined by visual evoked potentials (VEP), showing an enhancement in the latency and amplitude of recorded waves as a result of oral citicoline treatment [**7**].

The VEP examination is a useful tool for objectively quantifying the function of the visual system, being a diagnostic tool for neurological disorders. There are described two categories of VEP examination, Pattern and Flash. In the Pattern examination, the result is recorded as a succession of waves, N75 being the initial negative wave, followed by a positive wave, P100. The P100 wavelength or latency evaluation and the N75-P100 amplitude can be useful in monitoring visual dysfunction. Thus, increased latency of P100 and amplitude reduction are commonly found in glaucoma patients [**8**,**9**]. Therefore, improving these parameters of VEP waves by developing new therapeutic modalities is an objective in the management of glaucoma. In the case of Flash stimulation, changes of P2 wave were also noticed.

Optical Coherence Tomography (OCT) is a non-invasive method that allows the retinal nerve fiber layer (RNFL) thickness to be quantified by making sections at the optical disc level. Also, OCT can measure the RGC axons loss, another useful instrument for monitoring glaucoma progression [**10**]. The VEP and the OCT are powerful methods for glaucoma diagnosis. The sensitivity and specificity of these methods are similar in detecting early damage [**11**].

The goals of the current study were the following:

- Analysis of RGC layer and RNFL thickness evolution in patients receiving citicoline;

- The study of the P100 and P2 waves parameters evolution (amplitude, latency) during the citicoline treatment; 

- Determining correlations of the RGC layer or RNFL and the P100 and P2 waves parameters of the VEP examination.

## Materials and methods

The study is prospective, therapeutic, and includes 22 patients already having the diagnostic of primitive open-angle glaucoma (POAG). The conduction of this study and the tests, was performed in the Ophthalmology Clinic of the Bucharest Emergency University Hospital.

With the purpose to examine patients and to administer citicoline treatment, the patients signed the informed agreement. The research protocol was approved by the Ethics and Scientific Research Commission of “Carol Davila” University of Medicine and Pharmacy Bucharest (8218/ 15.02.2017).

The inclusion criteria in this study were the following:

- Patients of both sexes aged 18 to 75;

- Confirmed diagnosis of primitive open-angle glaucoma;

- IOP < 21 mmHg on unchanged topical hypotensive therapy for at least 3 months; 

- The reduction of RGCs on OCT.

- The exclusion criteria were the following:

- IOP > 21 mmHg;

- Optic neuritis;

- Macular or retinal disorders;

- Ophthalmologic surgery in the last 3 months;

- Hypersensitivity to citicoline;

- Diabetic patients, with multiple sclerosis, Parkinson’s disease, nystagmus.

The study group was formed by 22 patients (18 women and 4 males), aged 18 to 75 years, already diagnosed with POAG, and treated with local ocular hypotensive agents. A number of 43 eyes were analyzed because in one patient an eye was eliminated from the study, as the TIO was over 21 mmHg despite the hypotensive treatment.

Patient examination consisted of both clinical and paraclinical ophthalmologic investigations performed at the beginning of the study, before initiating general citicoline treatment (V0), then at 3 months (V1) and at 6 months (V2) from onset.

Clinical ophthalmologic examination performed at the beginning of the study, and then at 3 months, included visual acuity, IOP measurement, slit lamp inspection of the anterior pole, of the fundus, and gonioscopy.

Paraclinical investigations consisted of automatic perimetry examination, VEP, OCT with RNFL thickness measurement at the optic nerve level and RGC layer thickness.

For the VEP measurement, a Roland Consult (RETI-port MINIganzfeld I8) system was used. The testing was done monocularly, and two types of stimulation were performed, pattern and flash. For the pattern-VEP examination, the procedure consisted of placing the patient at a distance of 1 meter from the screen on which the stimulus was projected, consisting of a pattern similar to a reversible chess table with a frequency of 1/ second. Potential visuals were collected by the 3 electrodes fixed on the patient’s scalp, 12 cm above the nasion, at the vertex, 2 cm above the inion.

As a result of pattern stimulation, the recorded path was analyzed, thus the amplitude of the P100 wave was measured from the peak of negative N75 wave to the peak of the P100 positive wave. The latency was calculated from the moment of stimulus initiation to the top of each wave. The electrodes impedance was maintained below 5 kOhm. Flash-PEV stimulation is less sensitive than the pattern examination to the visual system dysfunction, and is mainly reserved for young, non-cooperating patients with media opacities or large refractive errors that cannot collaborate for pattern-PEV [**11**]. After the flash stimulation, the amplitude and latency of the resulting P2 wave were also analyzed. Investigation of VEP in the study consisted in performing both types of stimulation.

OCT was performed using a Carl Zeiss Cirrus HD-OCT 4000, thus measuring the RNFL thickness of the optic nerve and the RGCs layer thickness in the central macular area.

During the study, patients received each day a treatment consisting of 600 mg of citicoline, in the form of 4 capsules/ day (Neurovert-Sun Wave Pharma), each containing 150 mg of citicoline, plus Ginkgo biloba extract, Bacopa monnieri, alpha lipoic acid, salvia officinalis extract and phosphatidylserine.

After the initial clinical and paraclinical assessment by the above-mentioned methods, the treatment was started, and similar assessments were made at 3 months (V1) and 6 months (V2) from the beginning of the study.

***Statistical analysis***

It was performed using SPSS program (Windows). The outcomes were interpreted as medians. The effects of citicoline treatment on the studied parameters were statistically analyzed by Paired-Samples T-test, a value of p<0.05 guaranteeing a 95% confidence level of the results obtained.

## Results

Up to present, 22 glaucoma patients have been included in the research. Of these, 18% (4 patients) were male and 82% (18 patients) were female. The total number of eyes analyzed was 43 eyes, one eye being excluded because IOP > 21 mmHg.

Data analysis consisted of comparing the RGCs layer thickness of all eyes between successive visits and between the initial and the 6 months examinations. Following the same model, the parameters of the P100 and P2 waves were also studied, the amplitude increase and the decrease of the latency being considered positive outcomes of the citicoline treatment.

Analysis of RGCs layer through the Paired Samples Test indicated a small increase between successive visits of 1.53 μm between V0 and V1, 1.07 μm between V1 and V2 and 2.23 μm between V0 and V2 at 6 months, but the differences were not statistically significant (p> 0.05). The mean values of the RGCs later between visits showed a strong and positive correlation, which meant very small changes from one visit to another. The RGCs are neuronal cells and as we know their number could only be stable or decrease, thus the slight increase noted between visits was not clinically possible. This might be caused by an misidentification of the retinal layers in the previous examinations, as some authors have noted in their research [**12**].

The P100 wave amplitude analysis showed small variations from one visit to another by increasing or decreasing amplitude, but the correlations between these values were also strong and therefore could not be considered as the result of the treatment we applied.

Between the first visit and the 3 months visit, the amplitude of the P100 wave increased by an average of 0.02mV, between the 3 months and the 6 months visits there was an amplitude reduction of 1.06mV, and the contrast between the initial moment and V2 (6 months) indicated a grow of 2.98mV of P100 amplitude.

Latency analysis of P100 showed a slight increase of 0.68 ms at 3 months visit from baseline, followed by a reduction of 0.55ms to 6 months visit. A slight increase of 0.55 msec was found between the initial and the 6 months visit. There was a strong correlation between these data so that differences could not be considered statistically significant.

Regarding the Flash PEV examination, the amplitude, and latency of the recorded P2 wave was examined similarly to P100 wave at successive moments.

Thus, the amplitude of the P2 wave recorded a slight decrease of 0.37 mV at 3 months visit, then a rise of 0.33mV at 6 months, but the correlation between these successive moments was high, so neither of these scores was statistically significant. There were not noticed significant differences between the initial visit and the 6 months visit. The latency of P2 wave, recorded as a result of Flash PEV stimulation, increased between successive visits, with 2 ms between V0 and V1, and with 0.97 ms between V1 and V2, but the differences were not statistically significant (p > 0.05). The difference between P2 wave latency at initial and 6 months visits was 3.64 ms higher, and this was statistically significant (p = 0.01).

For each of the 3 moments of patient evaluation, we analyzed whether there were correlations between the thickness of RGC layer and the parameters of the VEP waves. Thus, on the initial visit (V0), statistically significant correlations were found between the RGCs layer and the P100 latency (p < 0.05) and between the amplitude of P100 and its latency, both correlations being negative, which meant that the evolution of a parameter was in opposite sense to each other. For example, decreasing the RGC layer thickness caused a rise in P100 latency (**Table 1**).

**Table 1:** Correlations between the RGCs layer, P100 wave amplitude and latency on the first visit

		Correlations		
		CG_V0	PEV_P100_AMP_V0	PEV_P100_LAT_V0
CG_V0	Pearson Correlation	1	0.244	-0.481**
	Sig. (2-tailed)		0.152	0.003
	N	39	36	36
PEV_P100_AMP_V0	Pearson Correlation	0.244	1	-0.600**
	Sig. (2-tailed)	0.152		0.000
	N	36	40	40
PEV_P100_LAT_V0	Pearson Correlation	-0.481**	-0.600**	1
	Sig. (2-tailed)	0.003	0.000	
	N	36	40	40
**. Correlation is significant at the 0.01 level (2-tailed).				

At the 3 months visit (V1), there was also a statistically significant negative correlation between the thickness of the RGC layer and the P100 latency in the sense of inverse change of one parameter related to the other. At the 6 months (V2) visit, there was a statistically significant positive correlation between the RGC layer and the P100 amplitude, so that high values of RGC layer correlated with high P100 amplitude values and vice versa (**Table 2**).

**Table 2:** Correlations between RGCs, P100 wave amplitude, and latency at the 6 months visit

		Correlations		
		CG_V2	PEV_P100_AMP_V2	PEV_P100_LAT_V2
CG_V2	Pearson Correlation	1	0.433**	-0.135
	Sig. (2-tailed)		0.007	0.418
	N	38	38	38
PEV_P100_AMP_V2	Pearson Correlation	0.433**	1	-0.284
	Sig. (2-tailed)	0.007		0.075
	N	38	40	40
PEV_P100_LAT_V2	Pearson Correlation	-0.135	-0.284	1
	Sig. (2-tailed)	0.418	0.075	
	N	38	40	40
**. Correlation is significant at the 0.01 level (2-tailed).				

Regarding the correlations existing between the thickness of the RGC layer and the P2 wave parameters P2 of the Flash PEV, at the time of the initial examination (V0), these were not noted. Instead, after the first 3 months of treatment at V1 visit, positive correlations (p = 0.04) were observed between the thickness of the RGCs layer and the amplitude of P2 wave, meaning that thick RGCs layer correlated with the high amplitude of P2 wave and vice versa (**Table 3**).

**Table 3:** Correlations between RGCs, P2 wave amplitude and latency at 3 months visit

		Correlations		
		CG_V1	PEV_P2_AMP_V1	PEV_P2_LAT_V1
CG_V1	Pearson Correlation	1	0.306*	0.179
	Sig. (2-tailed)		0.046	0.250
	N	43	43	43
PEV_P2_AMP_V1	Pearson Correlation	0.306*	1	0.182
	Sig. (2-tailed)	0.046		0.243
	N	43	43	43
PEV_P2_LAT_V1	Pearson Correlation	0.179	0.182	1
	Sig. (2-tailed)	0.250	0.243	
	N	43	43	43
*. Correlation is significant at the 0.05 level (2-tailed).				

The correlation is maintained 6 months after the onset of the study, the stable thickness of the RGC layer being correlated with a slight increase of the P2 amplitude (p = 0.019).

The RNFL analysis obtained by OCT of the optic nerve revealed a slight increase during our study. As previously mentioned, this data could be an examination artifact, because the RNFL can only be stable or decrease, as the neurons cannot increase their number after being lost [**12**]. Thus, at 3 months, an increase in RNFL thickness by an average of 1.58 μm was found, and it was statistically significant (p = 0.012). Between 3 and 6 months visit, the increase was only 0.889μm, but the result was not statistically significant (p > 0.05). The differences were more evident between the time of enrollment and the 6 months evaluation, when the RNFL thickness increased by an average of 2.447μm (p = 0.001). As this result could not be clinically possible, we could only conclude that the RNFL did not decrease during our treatment study (**Table 4**).

**Table 4:** Comparison between RNFL thicknesses at different examinations

					Paired Samples Test				
		Paired Differences		95% Confidence Interval of the Difference					
		Mean	Std. Deviation	Std. Error Mean	Lower	Upper	t	df	Sig. (2-tailed)
Pair 1	RNFL_OCT_V0 - RNFL_OCT_V1	-1.585	3.860	.603	-2.804	-0.367	-2.630	40	0.012
Pair 2	RNFL_OCT_V1 - RNFL_OCT_V2	-0.889	4.374	0.729	-2.369	0.591	-1.219	35	0.231
Pair 3	RNFL_OCT_V0 - RNFL_OCT_V2	-2.447	4.385	0.711	-3.889	-1.006	-3.441	37	0.001

The RNFL changes also correlated with the P100 and P2 waves’ parameters. Thus, the results showed a positive correlation between the thickness of the RNFL and the amplitude of the P100 (p = 0.022) and P2 (p = 0.007) waves at the beginning of the study. Low values of RNFL correlated with small amplitudes of these waves, and elevated RNFL values with high amplitudes of the 2 waves. At the same moment, negative correlations of RNFL thickness and P100 latency (p = 0.001) were observed, so that high RNFL values correlated with low latencies and vice versa. The correlation was positive (p = 0.016) between RNFL and wavelength P2 (**Table 5**).

**Table 5:** Correlations between RNFL layer, P100 and P2 amplitude and latency at the initial visit

			Correlations			
		RNFL_OCT_V0	PEV_P100_AMP_V0	PEV_P100_LAT_V0	PEV_P2_AMP_V0	PEV_P2_LAT_V0
RNFL_OCT_V0	Pearson Correlation	1	0.360*	-0.493**	0.415**	0.374*
	Sig. (2-tailed)		0.022	0.001	0.007	0.016
	N	43	40	40	41	41
PEV_P100_AMP_V0	Pearson Correlation	0.360*	1	-0.600**	0.463**	-0.058
	Sig. (2-tailed)	0.022		0.000	0.003	0.723
	N	40	40	40	40	40
PEV_P100_LAT_V0	Pearson Correlation	0-.493**	-0.600**	1	-0.297	-0.036
	Sig. (2-tailed)	0.001	0.000		0.063	0.826
	N	40	40	40	40	40
PEV_P2_AMP_V0	Pearson Correlation	0.415**	0.463**	-0.297	1	-0.073
	Sig. (2-tailed)	0.007	0.003	0.063		0.651
	N	41	40	40	41	41
PEV_P2_LAT_V0	Pearson Correlation	0.374*	-0.058	-0.036	-0.073	1
	Sig. (2-tailed)	0.016	0.723	0.826	0.651	
	N	41	40	40	41	41
*. Correlation is significant at the 0.05 level (2-tailed).						
**. Correlation is significant at the 0.01 level (2-tailed).						

The results also indicated a negative correlation between the amplitude of P100 at baseline and its latency (p = 0.00), which meant the decrease of latency with the increase of amplitude and vice versa.

Between the amplitude of the 2 waves, P100 and P2, there was also a positive correlation (p = 0.003) at the initial examination, in the sense of associating the high values of the 2 waves and the low ones.

The correlation between RNFL and the P100 amplitude disappeared (p> 0.05) after the first 3 months of citicoline treatment, but the negative correlation between RNFL and latency P100 (p = 0.005) and positive with the amplitude of P2 (p = 0.00) maintained.

Both at baseline and at 3 months, there was noticed a positive correlation between the RNFL measured on the OCT and P2 wave latency, which meant the correlation of elevated latency with those of RNFL and vice versa.

At 6 months (V2) from the beginning of the research, the results showed that the correlation between the RNFL thickness and the amplitudes of P100 (p = 0.013) and P2 (p = 0.023) waves was maintained. At the same moment of the study, there was a positive correlation between the magnitudes of the P100 and P2 waves (**Table 6**).

**Table 6:** Correlations between RNFL layer, P100 and P2 amplitude and latency at the 6 months visit

			Correlations			
		RNFL_OCT_V2	PEV_P100_AMP_V2	PEV_P100_LAT_V2	PEV_P2_AMP_V2	PEV_P2_LAT_V2
RNFL_OCT_V2	Pearson Correlation	1	0.400*	-0.207	0.582**	0.368*
	Sig. (2-tailed)		0.013	0.213	0.000	0.023
	N	38	38	38	38	38
PEV_P100_AMP_V2	Pearson Correlation	0.400*	1	-0.284	0.562**	-0.145
	Sig. (2-tailed)	0.013		0.075	0.000	0.371
	N	38	40	40	40	40
PEV_P100_LAT_V2	Pearson Correlation	-0.207	-0.284	1	-0.232	0.134
	Sig. (2-tailed)	0.213	0.075		0.149	0.411
	N	38	40	40	40	40
PEV_P2_AMP_V2	Pearson Correlation	0.582**	0.562**	-0.232	1	0.255
	Sig. (2-tailed)	0.000	0.000	0.149		0.112
	N	38	40	40	40	40
PEV_P2_LAT_V2	Pearson Correlation	0.368*	-0.145	0.134	0.255	1
	Sig. (2-tailed)	0.023	0.371	0.411	0.112	
	N	38	40	40	40	40
*. Correlation is significant at the 0.05 level (2-tailed).						
**. Correlation is significant at the 0.01 level (2-tailed).						

## Discussions

There is plenty of proof supporting the theory that neurodegeneration is the main pathogenic mechanism in glaucoma. Thus, although the primary stimulus is removed, glaucoma continues to progress [**13**]. P100 wavelength latency is thought to reflect both the function of retinal and post-retinal visual pathways. Consequently, nerve conduction impairment due to degeneration of neural fibers is responsible for increased PEV latency in glaucoma [**14**]. 

The effect of citicoline on VEP parameters, like increase of amplitude and decrease of latency was evidenced by *Rejdak*, also by oral administration of citicoline [**7**].

Electrophysiological tests are objective methods with reduced individual variability and can be used to study glaucomatous dysfunction. Paris also demonstrated the useful effect of the administration of citicoline on the parameters of the VEP [**15**].

The negative correlations noted in this study between CG and RNFL on the one hand and the latencies of PEV waves on the other hand were confirmed by *Esen* in patients with multiple sclerosis [**16**].

Unlike the results of the present study, where the correlations between P100 latency before and after treatment with citicoline were negative (signifying the increase in latency), *Rejdak* noticed a decrease in P100 latency, meaning a favorable outcome of the treatment [**7**].

The outcomes of a study conducted by Lee are similar to those obtained by us regarding the negative correlation of P100 latency with RNFL and the thickness of the CG layer. The same study also confirmed another result we obtained, the positive correlation of the RGC layer thickness with the amplitude of the P100 wave [**17**].

## Conclusions

The role of citicoline on the evolution of glaucomatous optic neuropathy is positive, as showed by the results, some of which are consistent with those obtained by other authors. The effects are mainly highlighted by functional VEP investigations, which indicate improvement of certain parameters. 

**Acknowledgements**

We would like to thank Sun Wave Pharma for their help during the study by providing free Neurovert for patient administration. We also thank Mrs. Raluca-Maria Bălă for supporting the statistical analysis of the study. All the authors had equal contribution to this paper.

**Disclosure statement**

None.
